# Ultrasound-guided bilateral superficial cervical plexus block enhances the quality of recovery of uremia patients with secondary hyperparathyroidism following parathyroidectomy: a randomized controlled trial

**DOI:** 10.1186/s12871-021-01448-w

**Published:** 2021-09-18

**Authors:** Shenghong Hu, Teng Shu, Siqi Xu, Xia Ju, Shengbin Wang, Li Ma

**Affiliations:** 1 Department of Anesthesiology, Anqing Hospital Affiliated with Anhui Medical University, No.352 Renmin road, Anqing, 246003 Anhui China; 2 Department of Thyroid and Breast Surgery, Anqing Hospital Affiliated with Anhui Medical University, No.352 Renmin road, Anqing, 246003 Anhui China

**Keywords:** Ultrasound-guided, Superficial cervical plexus block, Quality of recovery, Uremia, Secondary hyperparathyroidism, Parathyroidectomy

## Abstract

**Background:**

Parathyroidectomy has been proposed as a method for reducing parathyroid hormone levels. We evaluated the effects of ultrasound-guided bilateral superficial cervical plexus block (BSCPB) on the quality of recovery of uremia patients with secondary hyperparathyroidism (SHPT) following parathyroidectomy.

**Methods:**

Eighty-two uremia patients who underwent parathyroidectomy and exhibited SHPT were randomly allocated to the BSCPB group or the control group (CON group). The patients received ultrasound-guided BSCPB with 7.5 ml of ropivacaine 0.5% on each side (BSCPB group) or equal amount of 0.9% normal saline (CON group). The primary outcome of the Quality of Recovery-40(QoR-40) score was recorded on the day before surgery and postoperative day 1(POD1). Secondary outcomes including total consumption of remifentanil, time to first required rescue analgesia, number of patients requiring rescue analgesia, and total consumption of tramadol during the first 24 h after surgery were recorded. The occurrence of postoperative nausea or vomiting (PONV) and the visual analogue scale (VAS) scores were assessed and recorded.

**Results:**

The scores on the pain and emotional state dimensions of the QoR-40 and the total QoR-40 score were higher in the BSCPB group than in the CON group on POD1 (*P* = 0.000). Compared with the CON group, the total consumption of remifentanil was significantly decreased in the BSCPB group (*P* = 0.000). The BSCPB group exhibited longer time to first required rescue analgesia (*P* = 0.018), fewer patients requiring rescue analgesia (*P* = 0.000), and lower postoperative total consumption of tramadol during the first 24 h after surgery (P = 0.000) than the CON group. The incidence of PONV was significantly lower in the BSCPB group than in the CON group (*P* = 0.013). The VAS scores in the BSCPB group were lower than those in the CON group at all time-points after surgery (*P* = 0.000).

**Conclusion:**

Ultrasound-guided BSCPB with ropivacaine 0.5% can enhance the quality of recovery, postoperative analgesia, and reduce the incidence of PONV in uremia patients with SHPT following parathyroidectomy.

**Trial registration:**

ChiCTR1900027185 . (Prospective registered). Initial registration date was 04/11/2019.

## Background

Secondary hyperparathyroidism (SHPT) is a common disorder of calcium, phosphorus and vitamin D metabolism in patients with end-stage renal disease (ESRD) that results in mineral imbalance and bone disorders, especially in dialysis patients [1]. Various therapeutic agents, including phosphate binders, calcimimetics and less calcemic vitamin D analogs, can enhance the skeletal uptake of calcium and phosphate, resulting in remineralization and improved bone structure and strength [2, 3]. If parathyroid hormone levels are persistently elevated >800 pg/ml (>6 months) and pharmacological therapy remains ineffective, uremia patients with SHPT may undergo parathyroidectomy [4]. For total or subtotal parathyroidectomy, the surgical stimulus during gland dissection is mostly gentle, local or regional anesthesia is sufficient, and both types of anesthesia can provide postoperative analgesia [5–7]. However, the conventional anesthetic technique for parathyroidectomy is total intravenous anesthesia with tracheal intubation and muscle relaxation [8]. Additionally, ESRD affects the metabolism and excretion of opioid analgesics and muscle relaxants, which can prolong postoperative recovery [9, 10]. Furthermore, Uhlmann et al. [6] demonstrated that mild to moderate pain after parathyroidectomy may render patients ineligible for postoperative recovery programs. A previous study showed that two-thirds of patients undergoing parathyroidectomy require narcotic analgesics to relieve postoperative pain on the first day after surgery, whereas the incidences of postoperative nausea, vomiting and apnea or respiratory depression also increase [11–13].

Ultrasound-guided bilateral superficial cervical plexus block (BSCPB) could provide postoperative analgesia in thyroidectomy and parathyroidectomy. However,to date,the effects of ultrasound-guided BSCPB on the quality of recovery (QoR) following parathyroidectomy have not yet been evaluated. Therefore, the primary aim of this study was to investigate the effects of ultrasound-guided BSCPB on the QoR of uremia patients with SHPT following parathyroidectomy. The secondary objectives were to evaluate the postoperative analgesia and side effects of ultrasound-guided BSCPB.

Therefore, this study was conducted to examine the hypothesis that ultrasound-guided BSCPB would enhance the quality of recovery and postoperative analgesia while reducing the incidence of PONV in uremia patients with SHPT following parathyroidectomy.

## Methods

### Participants

This study was approved by the Ethics Committee of Anqing Hospital Affiliated with Anhui Medical University. The study was prospectively registered in the Chinese Clinical Trial Registry on November 4, 2019 (ChiCTR1900027185). Before surgery, each patient signed an informed consent form. The study was conducted at Anqing Hospital Affiliated with Anhui Medical University from November 2019 to October 2020.

Female or male uremia patients aged 18 and 65 years old of American Society of Anesthesiologists (ASA) physical status IV scheduled to undergo total parathyroidectomy were eligible. Patients were excluded if they had refusal to participate,platelet abnormalities, coagulation abnormalities, anticoagulation, serious cardiovascular and cerebrovascular diseases, hypertension (predialysis diastolic blood pressure, DBP > 110 mmHg), hyperkalemia (serum K^+^ concentration > 5.5 mmol/L), amino-amide local anesthetic allergy, local sepsis, and diaphragmatic motion abnormality. Diaphragmatic motion was evaluated using M mode sonography, and unilateral diaphragmatic motion was classified as normal, paretic, akinetic,and paradoxical [14].

The study was a randomized, double-blind, controlled clinical trial. All eligible participants were randomized to the BSCPB group or the CON group with a 1:1 allocation using computer-generated random numbers. Group assignments were kept in sealed envelopes, and only the nurse responsible for preparing the local anesthetic was allowed to open the envelope and the assigned drug. The assigned drugs according to group assignments in syringes had no difference in appearance. The patients and data collectors (anesthesiologists) did not know the drugs used for BSCPB. All the patients were nil per os (NPO)approximately 6 h before surgery.

### Study protocol

All surgeries were performed by three experienced surgeons. The patients were not given any preoperative medication. Dialysis was performed routinely within 24 h before surgery. The arteriovenous fistula of long-term dialysis patients were protected by medical staff during surgery. Noninvasive blood pressure (NBP), heart rate (HR), electrocardiogram (ECG) and peripheral pulse oximeter (SPO_2_) values were monitored using a multiparameter monitor (Philips MX500, Boeblingen, Germany). An intravenous catheter was inserted into the forearm without arteriovenous fistula, and Ringer’s solution was intravenously administered through the catheter at a rate of 3 ml/kg/h.

**Fig. 1:**
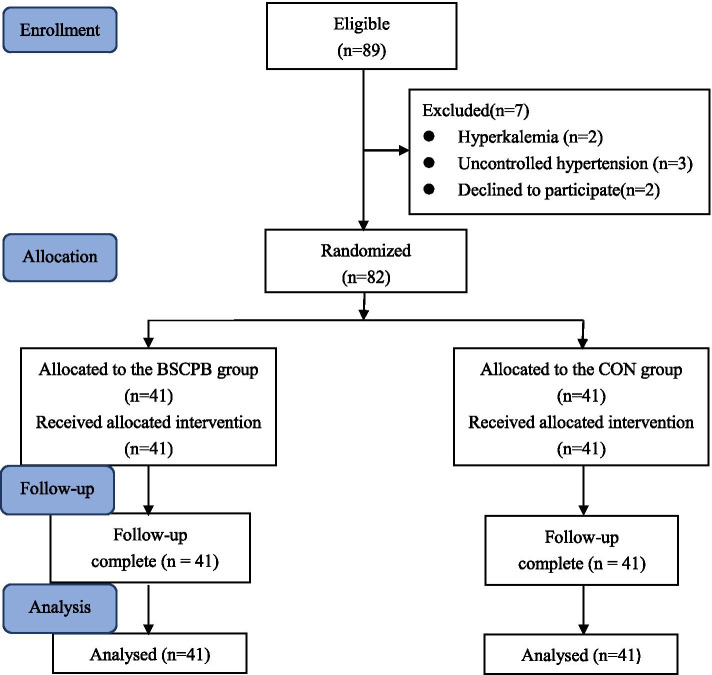
Ultrasound images of superficial cervical plexus in transverse view. CA: carotid artery; IJV: internal jugular vein; SCM: sternocleidomastoid muscle; SCP: superficial cervical plexus; C_4_: the fourth cervical vertebra. **A**:before block performance; **B**:after local anesthetics administration

As described by Gürkan et al. [15], all bilateral blocks were performed by the attending anesthesiologist who experienced techniques including ultrasound and nerve block before the induction of anesthesia. Patients were lying supine, and their heads were rotated to the opposite side of the block. The ultrasound probe and the skin of the blocked area were routinely sterilized. A high-frequency linear ultrasonic probe (SonoSite Turbo; SonoSite Inc., Bothell, Wash, USA) was placed on the posterior margin of the sternocleidomastoid (SCM) muscle at the level of fourth cervical vertebra(C_4_). At the posterior corner of the SCM muscle, the superficial cervical plexus was visualized as a hypoechoic structure (Fig. 1). The 22-gauge needle was inserted under the SCM muscle using the in-plane technique. The needle position and location were confirmed by injecting 0.5–1 ml of solution after negative aspiration of no blood and air, 7.5 ml ropivacaine 0.5% was administered on each side in the BSCPB group, while equal amount of 0.9% normal saline was administered in the CON group (Fig. 1). After being administered, no block-related side effects, such as anesthetic toxicity, epidural block anesthesia, total spinal block anesthesia, recurrent laryngeal nerve blockade, phrenic nerve blockade or Horner’s syndrome, were confirmed, and general anesthesia was induced.

General anesthesia was induced with midazolam (0.02 mg/kg), propofol (2.0 mg/kg), sufentanil (0.3 μg/kg) and cisatracurium (0.15 mg/kg). After adequate muscle relaxation, an endotracheal tube containing surface electrodes (NIM-Response Nerve Integrity Monitoring, Medtronics) was inserted using a video laryngoscope (Youyi Medical,Zhejiang,China),and surface electrodes were positioned at the glottic level in contact with both true vocal folds [16]. All patients were ventilated with an Aspire View anesthetic machine (GE Healthcare, Madison, WI, USA) in volume control mode. In the two groups, the tidal volume (VT) was maintained at 8–10 ml/kg, respiratory rate (RR) was fixed at 10–12 breaths/min, inspiratory-to-expiratory-time ratio (I:E) was 1:2 and inspired oxygen fraction (FiO_2_) was 0.5 (balanced with air) throughout the anesthesia period. After administration of induction doses of muscle relaxants, no muscle relaxants were administered during surgery. Anesthesia was maintained with target-controlled infusions of propofol (effect-site concentration, Ce 2–4 μg/ml) and remifentanil (Ce 2–8 ng/ml) using closed-loop titration guided by bispectral index (BIS, GE Healthcare, Madison, WI, USA) [17]. BIS values was maintained between 45–60 to prevent the occurrence of intraoperative awareness. The medication was administered manually or switched to manual infusion during surgery if required. Experienced surgeons preserved the anatomical integrity of motor nerves by visual identification and exposure of both the external branch of the superior laryngeal nerve and the recurrent laryngeal nerve. Additionally, the recurrent laryngeal nerve prevented injury by intraoperative neuromonitoring during parathyroidectomy.

IV bolus infusion of tolansetron 4 mg was used to prevent postoperative nausea or vomiting (PONV) at the end of surgery. All patients retained the endotracheal tube and were transferred to the postanesthesia care unit (PACU). The endotracheal tube was removed after full recovery of consciousness and spontaneous ventilation, and the train-of-four ratio was at least 0.9. If the Steward recovery score was >4 points [18], the patient escorted back to the ward from the PACU. The administration of postoperative routine analgesia was IV bolus infusion of parecoxib sodium 40 mg at the end of surgery, followed by an IV bolus infusion of parecoxib sodium 40 mg every 12 h for the next 24 h. If the visual analog scale (VAS) scores was at least 3 or the patient requested analgesia, IV bolus infusion of tramadol 50 mg was given as a rescue analgesic.

### Data collection

Demographic and clinical characteristics, including age, height, weight,gender, HGB (hemoglobin, preoperative and within 10 min after surgery), PLT (platelets), APTT (activated partial thromboplastin time), PT (prothrombin time), TT (thrombin time), and Fib (fibrinogen), were recorded. Additionally, the duration of anesthesia and surgery was documented. Intraoperative fluid input was recorded. Intraoperative blood loss was recorded and evaluated by the HGB value, which was measured preoperatively and within 10 min after surgery [19]. Each patient was assessed using the global QoR-40 score [20] on the day before surgery and POD1. The QoR-40 includes five dimensions: emotional state (9 items), physical comfort (12 items), physical independence (5 items), psychological support (7 items) and pain (7 items). Each item is assessed using a 5-point numerical rating scale. The QoR-40 score ranges from 40 to 200. The recovery state is proportional to the score (40 = extremely poor recovery, 200 = excellent recovery). The total consumption of remifentanil was recorded. For the first time to required rescue analgesia, number of patients requiring rescue analgesia and total consumption of tramadol during the first 24 h after surgery were recorded. The total number of patients who vomited and the number of vomits were recorded. The occurrence of PONV was assessed by the PONV Intensity Scale, and PONV Intensity Scale score of 50 was defined as clinically significant PONV [21]. The VAS scores at 2, 4, 8, 12 and 24 h after surgery were rated using a 10-cm visual analog scale (VAS: 0 = no pain, 10 = the most pain imaginable). Block-related side effects were observed.

### Statistical analysis

The calculation of sample size was based on the global QoR-40 score. A change of 10 points or more on the QoR-40 signifies a clinically important difference. In our pilot study, an overall standard deviation of 13 points was estimated, and with an α of 0.05, 37 patients would be required in each group (assuming a power of 80%). Anticipating a study drop-out rate of 10%, we included 41 patients per group.

Data analysis was performed using SPSS version 23.0 (SPSS Inc., Chicago, IL). Shapiro-Wilk test was used to evaluated the normal distribution of data. Continuous variables were presented as the mean (standard deviation, SD). Normally distributed variables were compared using Student’s t-test. Nonnormally distributed variables were presented as the median (interquartile range, IQR), and compared using the Mann–Whitney U test. VAS scores was compared using repeated- measures analysis of variance, and post hoc multiple comparisons was performed with Bonferroni correction. Qualitative data was presented as number or percentage, and compared using the chi-square test. *P* values of less than 0.05 was considered to be statistically significant.

## Results

**Fig. 2:**
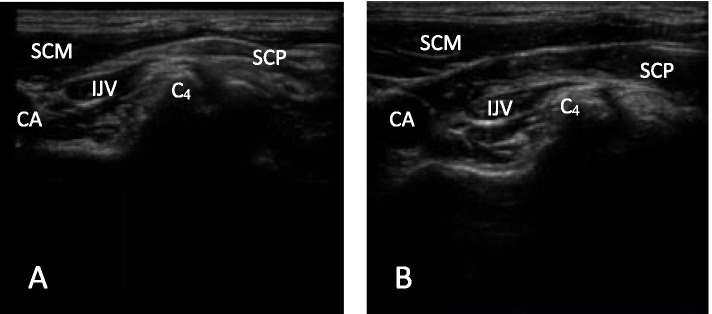
CONSORT flow diagram for the study

**Fig. 3:**
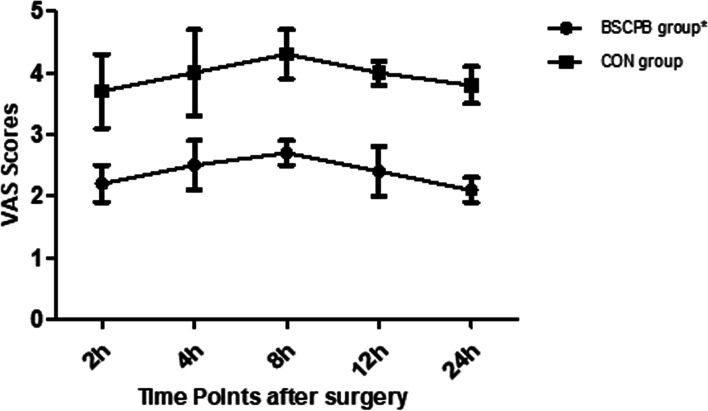
Categorical variables were presented as the mean(SEM) for all of the patients, with 41 cases in each group.*P=0.000 different from the CON group at all time-points after surgery. SEM: standard error of mean; BSCPB: bilateral superficial cervical plexus block; CON: control

A total of 89 patients were assessed for eligibility for the study, and 82 subjects were enrolled in the study (Fig. 2). Seven patients were excluded (reasons for exclusion were listed in Fig. 2). There were no significant differences in the groups with respect to age, height, weight, gender, HBG (preoperative or within 10 min after surgery), PLT, APTT, PT, TT or Fib (Table 1). There were no significant differences in the groups with respect to intraoperative fluid input, intraoperative blood loss, duration of anesthesia or duration of surgery (Table 2). As shown in Table 3, the score on the pain dimension (27.0 vs. 23.0, *P* = 0.000) and the emotional state dimension (38.0 vs. 33.0, *P* = 0.000) of the QoR-40 and the total QoR-40 score (162.0 vs. 149.0, *P* = 0.000) were higher in the BSCPB group on POD1. Compared with the CON group, the total consumption of remifentanil was significantly decreased in the BSCPB group (P = 0.000) (Table 2). The BSCPB group exhibited longer time to first required rescue analgesia (15.4 h vs. 8.3 h, *P* = 0.018) and fewer patients requiring rescue analgesia (20/41 vs. 36/41, *P* = 0.000) than the CON group. Furthermore, postoperative total consumption of tramadol during the first 24 h after surgery was lower (*P* = 0.000) (Table 4). The incidence of PONV was significantly lower in the BSCPB group (4.9%) than the CON group (24.4%) (P = 0.013) (Table 4). The VAS scores in the BSCPB group were lower than those in the CON group at all time-points after surgery (P = 0.000) (Fig. 3). No block-related side effects, such as anesthetic toxicity, epidural block anesthesia, total spinal block anesthesia, recurrent laryngeal nerve blockade, phrenic nerve blockade or Horner’s syndrome, were observed.

**Table 1:** Demographic and laboratory results

Variables	BSCPB group (*n*=41)	CON group (*n*=41)
Age (yr)	54.5 (8.1)	53.4 (6.1)
Height (cm)	156.7 (5.6)	155.9 (6.3)
Weight (kg)	57.8 (6.5)	61.1 (5.4)
Gender, Female/Male(n)	25/16	21/20
HGB(g/L) (preoperative)	106.4 (15.5)	102.6 (15.6)
HGB(g/L) (within 10min after surgery)	103.5 (14.3)	100.9 (14.8)
PLT(10×10^9^/L)	166.4 (39.6)	161.8 (45.5)
PT(s)	10.8 (1.2)	11.1 (1.5)
APTT(s)	29.7 (5.4)	31.2 (4.2)
TT(s)	17.6 (0.8)	17.3 (1.3)
Fib(g/L)	3.2 (0.7)	3.2 (0.6)

Categorical variables were expressed as the mean (SD) or number for all of the patients, with 41 cases in each group. *BSCPB* Bilateral superficial cervical plexus block, *CON* Control.

**Table 2:** Clinical profile during surgery

Variables	BSCPB group (*n* =41)	CON group (*n* =41)	*P* value
Duration of anesthesia (min)	96.1 (24.3)	90.0 (25.6)	0.551
Duration of surgery(min)	78.2 (22.3)	70.1 (20.9)	0.385
Intraoperative fluid input(ml)	166.9 (49.1)	165.0 (45.9)	0.968
Intraoperative blood loss(ml)	14.6(5.7)	15.0 (5.2)	0.876
Total consumption of remifentanil (micro g)	533.4 (59.9)	580.1 (76.1)	0.000

Categorical variables were expressed as the mean (SD) for all of the patients,with 41 cases in each group. *BSCPB* Bilateral superficial cervical plexus block, *CON* Control.

**Table 3:** Preoperative and POD1 Global QoR-40 Score

	BSCPB group(n=41)	CON group(n=41)	*P-*value
Preoperative			
Total QoR-40 Score	156.0 (148.5 to164.0)	154.0 (147.0 to 161.5)	0.452
POD1			
Emotional state	38.0 (35.0 to 40.0)	33.0 (31.0 to 35.0)	0.000
Physical comfort	46.0 (43.0 to 49.0)	44.0 (41.5 to 46.0)	0.076
Physical independence	21.0 (19.0 to 22.0)	20.0 (19.0 to 21.5)	0.614
Psychological support	30.0 (29.0 to 32.0)	29.0 (28.0 to 31.5)	0.055
Pain	27.0 (25.0 to 29.0)	23.0 (20.0 to 25.0)	0.000
Total QoR-40 Score	162.0 (157.5 to166.5)	149.0 (144.0 to153.5)	0.000

Categorical variables were presented as the median (IQR) for all of the patients, with 41 cases in each group. *BSCPB* Bilateral superficial cervical plexus block, *CON* Control, *POD1* Postoperative day 1.

**Table 4:** Recovery profile after surgery

Variables	BSCPB group (*n*=41)	CON group (*n*=41)	*P* value
Time to first require rescue analgesia (h)	15.4 (1.9)	8.3 (1.2)	0.018
Number of patients requiring analgesia(n,%)	20 (48.8%)	36 (87.8%)	0.000
Postoperative tramadol consumption(mg)	24.4 (25.3)	43.9 (16.6)	0.000
Incidence of PONV(n,%)	2 (4.9%)	10 (24.4%)	0.013

Categorical variables were presented as the mean (SD) or number (proportion) for all of the patients, with 41 cases in each group; *BSCPB* Bilateral superficial cervical plexus block, *CON* Control.

## Discussion

This study demonstrated that ultrasound-guided BSCPB with ropivacaine 0.5% combined with general anesthesia was effective in enhancing recovery quality compared with single general anesthesia in uremia patients with SHPT following parathyroidectomy. Ultrasound-guided BSCPB could reduce the intraoperative total consumption of remifentanil, provide satisfactory analgesic effects within 24 h after surgery, and reduce the incidence of PONV. Furthermore, no block-related side effects were observed.

General anesthesia can provide satisfactory anesthetic effects while eliminating operative anxiety [22], but local/regional anesthesia can reduce the total consumption of opioid analgesics during surgery and lower postoperative pain [23]. Thus, regional anesthesia combined with general anesthesia was chosen as anesthetic technique in this study.

The QoR-40 scale has been widely used and extensively validated to assess the quality of postoperative recovery through specific and patient-rated questionnaires [20, 24]. Myles et al. [25] pointed out that a change of 6.5 points or more in QoR-40 score signifies an important difference, indicating a clinically relevant improvement in the quality of recovery after surgery. In our pilot study, we demonstrated a 13-point difference in QoR-40 score at POD1. Therefore, our study indicated that ultrasound-guided BSCPB with ropivacaine 0.5% could improve the postoperative health status of uremia patients with SHPT following parathyroidectomy.

Patients experience mild to moderate postoperative incision site pain after thyroidectomy or parathyroidectomy. Satisfactory postoperative analgesia can improve the postoperative emotional state [26]. A previous study showed that postoperative pain strongly affects the score on the pain and physical comfort dimension of the QoR-40 [27]. In our study, the results showed that the scores on the pain and physical comfort dimension of the QoR-40 were higher in the BSCPB group than in the CON group on POD1. We speculated that the higher score on the emotional state dimension in the BSCPB group than in the CON group might be attributed to the higher score on the pain dimension in our study. Thus, the results of this study inferred that effective bilateral cervical plexus block could improve the pain and physical comfort dimension of the QoR-40 after parathyroidectomy surgery.

Currently, valid measures of pain intensity include Numerical Rating Scale (NRS), VAS, Verbal Rating Scale (VRS) and Faces Pain Scale-Revised (FPS-R) [28]. The reliability of the VAS for postoperative acute pain assessment was high, and as described by Bijur et al. [29], in the early stage of this study, the reproducibility of the VAS was verified by paired VAS measurements obtained 1 min apart every 30 min over two hours. An important finding was observed in this study: the VAS scores was lower in the BSCPB group than in the CON group within 24 h after surgery, and the number of patient requiring rescue analgesia in the BSCPB group was smaller than that in the CON group. Therefore, the results of the study indicated that BSCPB could offer better analgesic efficacy in the early postoperative period after surgery, which was in agreement with a previous study [30].

Opioid analgesics are prescribed to relieve pain [5]. However, inappropriate opioid usage patterns can increase drug-related adverse effects, increase the risk for dependence and abuse, increase the incidence of PONV and delay postoperative recovery [24, 31].Long-acting nerve block can minimize postoperative opioid use [32]. In this study, another important finding was observed that the opioid-sparing properties and better analgesia obtained by preoperative BSCPB,which resulted in significantly lower incidence of PONV in the BSCPB group than in the CON group.

Compared with landmark-based technology, ultrasound-guided technology significantly improved the success rate of nerve block and reduced the incidence of block-related side effects, mainly because ultrasound-guided technology enables real-time visualization of the anatomical structures, advancement of the needle within tissues and distribution of the local anesthetic in the tissue during nerve block [33]. Therefore, no block-related side effects were observed in our study.

There were several limitations in this study. First, the length of stay in the PACU and postoperative length of hospital stay were not recorded, although two indexes are traditional parameters to assess postoperative recovery [34]. Second, despite multimodal analgesia protocol could relieve postoperative acute pain, and reduce the consumption of opioid and the incidence of related adverse effects [35], multimodal analgesia regimen was not used in this study. So the generalizability of the results was limited. Third, end-stage renal disease can affect left ventricular function [36], but mortality and cardiovascular events were not evaluated. Fourth,after parathyroidectomy, hypocalcemia may occur after surgery [37]. However, the effects of hypocalcemia on postoperative recovery were not evaluated. Finally, this study was a single-center clinical study, and the conclusions still need to be further supported by studies with large samples and multicenter studies.

## Conclusions

Compared with single general anesthesia, ultrasound-guided BSCPB with ropivacaine 0.5% combined with general anesthesia is effective in enhancing the quality of recovery, reducing the intraoperative consumption of remifentanil, providing satisfactory analgesia within 24 h after surgery, and decreasing the incidence of PONV in uremia patients with SHPT following parathyroidectomy. Furthermore, no block-related side effects were observed.

## Data Availability

The datasets used and/or analyzed during the current study are available from the corresponding author (Shengbin Wang, shbw1965@yeah.net) on reasonable request.

## Abbreviations

SHPT: Secondary Hyperparathyroidism
ESRD: End-Stage Renal Disease
ASA: American Society of Anesthesiologists
DBP: Diastolic Blood Pressure
POD: Postoperative Day
CON: Control
BSCPB: Bilateral Superficial Cervical Plexus Block
NBP: Noninvasive Blood Pressure
HR: Heart Rate
ECG: Electrocardiogram
SPO_2_: Peripheral Pulse Oximeter
VT: Tidal Volume
RR: Respiratory Rate
FiO_2_: Inspired Oxygen Fraction
Ce: Effect-Site Concentration
BIS: Bispectral Index
SCM: Sternocleidomastoid
PONV: Postoperative Nausea or Vomiting
PACU: Postanesthesia Care Unit
VAS: Visual Analogue Scale
HGB: Hemoglobin
PLT: Platelets
PT: Prothrombin Time
APTT: Activated Partial Thromboplastin Time
TT: Thrombin Time
Fib: Fibrinogen
IQR: Interquartile Range
NRS: Numerical Rating Scale
VRS: Verbal Rating Scale
FPS-R: Faces Pain Scale-Revised
